# Advances in High-Resolution Radiation Detection Using 4H-SiC Epitaxial Layer Devices

**DOI:** 10.3390/mi11030254

**Published:** 2020-02-28

**Authors:** Krishna C. Mandal, Joshua W. Kleppinger, Sandeep K. Chaudhuri

**Affiliations:** Department of Electrical Engineering, University of South Carolina, Columbia, SC 29208, USA; jwk@email.sc.edu (J.W.K.); chaudhsk@mailbox.sc.edu (S.K.C.)

**Keywords:** silicon carbide, 4H-SiC, epitaxial layer, Schottky barrier, radiation detector, point defects, deep level transient spectroscopy (DLTS), thermally stimulated current spectroscopy (TSC), electron beam induced current spectroscopy (EBIC), pulse height spectroscopy (PHS)

## Abstract

Advances towards achieving the goal of miniature 4H-SiC based radiation detectors for harsh environment application have been studied extensively and reviewed in this article. The miniaturized devices were developed at the University of South Carolina (UofSC) on 8 × 8 mm 4H-SiC epitaxial layer wafers with an active area of ≈11 mm^2^. The thicknesses of the actual epitaxial layers were either 20 or 50 µm. The article reviews the investigation of defect levels in 4H-SiC epilayers and radiation detection properties of Schottky barrier devices (SBDs) fabricated in our laboratories at UofSC. Our studies led to the development of miniature SBDs with superior quality radiation detectors with highest reported energy resolution for alpha particles. The primary findings of this article shed light on defect identification in 4H-SiC epilayers and their correlation with the radiation detection properties.

## 1. Introduction

Development of advanced high-resolution radiation detectors is aimed at those that are capable of reliable and long-term non-degrading operation at elevated temperatures under high doses of ionizing radiation. The application of such radiation detectors is primarily in the field of, but not restricted to, biomedical and nuclear engineering, medical imaging devices, and homeland security including nuclear materials accounting and safeguarding in harsh environments. Hence, to meet the criteria of field deployment, such detectors need to be compact and miniaturized. Applicability of high-resolution germanium (Ge) or silicon (Si) based detectors are extremely limited in these fields owing to their cryogenic operating temperature (low bandgap energy) and lack of radiation hardness. Room temperature compact detectors like cadmium telluride (CdTe) or cadmium zinc telluride (CdZnTe, CZT) falls behind when it comes to high temperature applicability as these materials cannot survive high temperatures. Silicon carbide (SiC), a wide band-gap semiconductor, has emerged as a potential alternative to more mature technologies in intense and rugged environments. Due to its wide bandgap (3.27 eV at 300 K), 4H-SiC devices show extremely low reverse leakage current at room temperature and elevated temperatures as well [1,2,3]. Detectors based on 4H-SiC epitaxial layers with low defect densities and impurities can be used to reliably detect any type of ionizing radiation at high radiation load (dose level of 22 MGy and higher [4,5]), in conditions of aggressive medium and at elevated temperatures, in systems for monitoring in acid/alkaline-containing media, and in systems that are designed for accurately determining the field of ionizing particles, X- and γ-ray radiation. SiC does not melt under laboratory conditions but sublimes at temperatures as high as 2700 °C and is highly chemically inert. Polytype 4H-SiC has a very high threshold displacement energy (22–35 eV) which accounts for its extreme radiation hardness [6,7]. Over the past decade, the quality of SiC material has improved significantly, which added momentum to the development of SiC based devices. Recent availability of high quality 4H-SiC epitaxial layers has oriented the radiation detector community to use such epilayers for radiation detection measurements with highly promising results. Although the resolution of the detectors based on bulk semi-insulating (SI) SiC grown by physical vapor transport (PVT) is not adequate, presumably due to high density of defects and deep level centers [7], the Schottky barrier device (SBD) detectors fabricated using SiC epitaxial layers [8,9,10] perform extremely well in high-resolution detection of low penetration depth radiation. Additionally, the response of these kind of detectors for soft X-rays are significantly higher than that of commercial off-the-shelf (COTS) SiC UV photodiode. 

The present article covers our recent results and reviews the advances towards the goal of achieving high-resolution and compact radiation detectors to be used in harsh environmental conditions. The majority of the developments in this article were carried out at Photovoltaics and Nuclear Radiation Accounting Devices (PANRAD) laboratory at the University of South Carolina (UofSC). Devices on 4H-SiC epitaxial layers were fabricated solely in our laboratory and characterized in terms of physical properties, epitaxial layer quality, electrical and radiation detection properties, and defect levels’ identification and quantification. The article focusses on the investigation of electrical properties like electron-hole pair creation energy and minority carrier diffusion length, and device properties such as surface barrier height, built-in potential, and leakage current. Point and structural defects that limit device performance were discussed in detail in the various types of epitaxial layers through defect delineating etching and XRD rocking curve measurements, electron beam induced current spectroscopy (EBIC), thermally stimulated current (TSC) spectroscopy, and deep level transient spectroscopy (DLTS). Electronic noise characteristics of the associated front-end electronics which affect the radiation detection properties of 4H-SiC for high-energy alpha particles were also investigated and presented. We also cover pulse height spectra (PHS) with 5486 keV alpha particles and 59.6 keV γ-rays from radiation sources, and soft X-ray responsivity measurements performed at the National Synchrotron Light Source (NSLS) at Brookhaven National Laboratory (BNL).

## 2. Materials and Methods 

**Figure 1:**
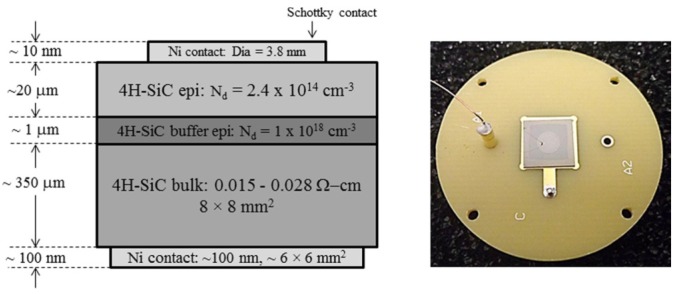
A cross-sectional schematic view of the epitaxial layer structure and a single pixel n-type Ni/4H-SiC Schottky barrier device (SBD) mounted on a printed circuit board (PCB). The square shaped back contact is visible through the wafer. The circular contact on the top is connected using a 25 µm thin gold wire.

In this article we present the results obtained from 20 and 50 μm thick n-type epitaxial layer grown on 76 mm diameter 4H-SiC (0001) wafer, which was highly doped with nitrogen and off-cut 8° towards the [11$\bar{2}$0] direction. The typical effective doping concentration in these epitaxial layers measured using high frequency (100 kHz) capacitance-voltage (C-V) method was found to be 1.8 × 10^14^–3.2 × 10^14^ cm^−3^. The radiation detectors were fabricated on 8 × 8 mm^2^ substrates diced from the 76 mm diameter wafer by depositing 3.2–3.8 mm in diameter and ≈10 nm in thickness Ni Schottky contacts on top of the epitaxial layers through a shadow mask and using a Quorum model Q150T sputtering unit. Large Ni contacts (≈6 × 6 mm^2^) of ≈100 nm in thickness were deposited on the other side of the wafer by the same means. A schematic of a typical cross-sectional view of the epitaxial layer with nickel contacts structure is shown in Figure 1.

For the semi-insulating (SI) 4H-SiC detector fabrication, ≈390 μm thick 8 × 8 mm^2^ substrates, diced from (0001) 76 mm semi-insulating wafer with resistivity ≥ 10^12^ Ω-cm, were used. The electrical contacts on the test structures were achieved by electron-beam deposition of 3.2 and 1.2 mm diameter Ni contacts on the Si face of the substrate and 3.2 mm in diameter or larger size (≈6 × 6 mm^2^) Ni contact onto the C face of the substrate using appropriately designed shadow masks. The substrates were cleaned using a standard Radio Corporation of America (RCA) cleaning procedure [11] prior to contact deposition. No annealing treatment was conducted after the deposition of Ni contacts unless otherwise mentioned in this article. For the high performing samples, the RCA cleaning of the wafers and the electrical contact deposition were achieved using photolithography techniques performed in class 100 clean room facilities. It may be noted here that the thickness of the Ni contact plays a pivotal role in defining the energy resolution of the detector. Thicker contacts may add to the variation of the incident energy due to excessive scattering. We deposited Ni contacts as thin as possible and simultaneously inspected whether the contact produced reliable electrical contacts or not. We adopted the minimum thickness as the optimized thickness of detector window, which produced reliable contacts confirmed from repeated I-V measurements.

A wire bonding technique was developed in our laboratory for achieving strong and durable electrical connections with the Ni contacts and minimizing the bonding area for efficient radiation detection. This technique involves special type of silver epoxy rated for high temperature and vacuum applications. The same type of the silver epoxy was used for mounting the chip on a printed circuit board (PCB). A photograph of a typical single pixel detector is shown in Figure 1.

TSC measurements on the epitaxial layer were conducted in the temperature range 94–550 K in vacuum < 1 × 10^−4^ Torr at 4–15 K/min heat rates. The trap filling was achieved by illuminating the samples at 94 K using UVP model UVM-57 Handheld UV Lamp specified to produce 302 nm UV light. The detailed description of our TSC measurement set-up is available in our earlier work [12].

Current-voltage (I-V) characterizations were performed using a Keithley (Cleveland, OR, USA) 237 high voltage source-measure unit. For the low temperature measurements, a vacuum cryo-chamber from MMR Technologies (CA, USA) was used. Capacitance-voltage (C-V) measurements were performed using either a Keithley (Cleveland, OR, USA) 590 CV analyzer or the DLTS measurement unit.

The DLTS measurements were carried out using a SULA (Ashland, OR, USA) DDS-12 modular DLTS system. The DLTS system comprised of a pulse generator module for applying repetitive bias pulses, a 1 MHz oscillator for capacitance measurements, a sensitive capacitance-meter involving self-balancing bridge circuit, and a correlator/pre-amplifier module which automatically removes DC background from the capacitance meter and amplifies the resultant signal change. The correlators were based on a modified double boxcar signal averaging system. The sample was mounted in a Janis (Woburn, MA, USA) VPF 800 LN2 cryostat for temperature variation, which was controlled by a Lakeshore (Cleveland, OH, USA) LS335 temperature controller. The DDS-12 system allows the user to collect four DLTS spectra simultaneously corresponding to four different rate windows in a single temperature scan. The signals were digitized using a national instruments (NI, Austin, TX, USA) digitizer card integrated with the DLTS system for on line processing using a personal computer (PC). The entire system including the modules and the temperature controller is controlled using a dedicated LabVIEW interface, which also allows the user to analyze the recorded data.

In order to evaluate the density of crystallographic defects, defect delineating chemical etching in molten KOH was performed at ≈825 K for about 5 min. Threading edge, screw, and basal plane dislocation densities (BPDs) were assessed via etch pit density (EPD) measurements using a Nomarski optical microscope (Make and model). X-ray diffraction rocking curves were acquired using a double crystal diffractometer (DSO-1), by Radicon Scientific Instruments Co. (Pune, India). We used CuK_α_ radiation and (0008) reflection in the rocking curve measurements. The EBIC measurements were carried out at 29 kV accelerating voltage and 0 V bias voltage using a JEOL (Peabody, MA, USA) 35 SEM scanning electron microscope (SEM).

A vacuum annealing apparatus was specially designed in our laboratory for the isochronal annealing studies. The annealing set-up comprised of a furnace with a temperature control unit, an oil-free mechanical pump, a turbo-molecular pump, vacuum gauge controllers, and turbo pump controller. The samples were loaded in quartz ampoules and placed under vacuum on the order of 2 × 10^−7^ torr. Upon reaching the desired temperature, the sample under vacuum was lowered to the hot zone of a single-zone tubular furnace. The samples were annealed for a duration of ≈30 min. Before each annealing stage, the top and bottom nickel contact of the Schottky devices were completely etched-off using concentrated nitric acid (HNO_3_). After annealing at each temperature, the samples were cleaned by RCA cleaning procedure and Ni Schottky and Ohmic contacts were deposited for subsequent I-V, C-V, and DLTS measurements. 

Pulse height spectroscopy (PHS) was carried out using a spectrometer comprising of a pre-amplifier (Cremat, Newton, MA, USA), CR-110 or Amptek (Bedford, MA, USA) A250 CoolFET) whose signals were fed to an Ortec (Oak Ridge, TN, USA) 572 spectroscopy amplifier. The shaped signals were digitized and binned to obtain pulse-height spectra using a Canberra (Yvelines, France) Multiport II multichannel analyzer (MCA) unit, controlled by Genie 2000 (Canberra (Yvelines, France)) interface software. Pulse-height spectra of 5486 keV alpha particles and 59.6 keV gamma rays were obtained using a ≈1.0 µCi ^241^Am (alpha and X-γ ray) radiation source. The source and the detector were placed inside an electromagnetic interference shielded aluminum box, which was constantly evacuated during the data acquisition using a vacuum pump, in order to minimize scattering of alpha particles with air molecules. 

The detectors fabricated in our laboratory at UofSC using n-type 4H-SiC epitaxial layers were also tested and evaluated at Los Alamos National Laboratory (LANL, Los Alamos, NM, USA) for detecting low energy X-rays and compared to commercial-off-the-self (COTS) SiC UV photodiode detectors. The measurements were performed at 20-250 V bias voltages using U3C and X8A beam lines [13] at the National Synchrotron Light Sources (NSLS) at Brookhaven National Laboratory (BNL, New York, NY, USA). This beam line provides monochromatic photon beams ranging from 50 to 6500 eV with intensities as high as 10^12^ photons/second.

## 3. Results

The following results were obtained using various 4H-SiC epitaxial layer detectors fabricated at UofSC. The wafers were categorized based on their performance as radiation detectors and are tabulated below in Table 1.

### 3.1. Electrical Measurements on SI and n-Type Epitaxial Layers

**Figure 2:**
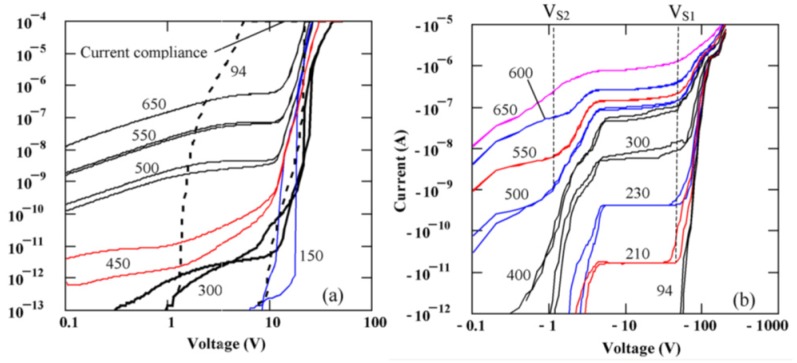
Forward (**a**) and reverse (**b**) I-V characteristics obtained for SI 4H-SiC sample (SI-M50) with 1 mm diameter Ni contact at various temperatures.

Figure 2 represents the I-V characteristics for semi-insulating 4H-SiC epitaxial (SI-M50) samples [14]. It can be observed from the graphs that the I-V characteristics are asymmetric with respect to the polarity of the applied bias. Currents higher than 10 µA at room temperature were observed for positive bias as low as 20 V on the Ni/SI-SiC surface. As a result, a positive bias will be referred to as the forward bias henceforth. The asymmetry of the I-V curves are thought to be due to the different potential distribution at the Ni/SI-SiC and SI-SiC/n^+^SiC interfaces [14]. It could also be observed from Figure 2 that each I-V curve at a given temperature in the forward bias region consists of two branches corresponding to ramping the bias voltage up and down showing a hysteresis. The difference is pronounced for temperatures below 600 K. Further, the reverse I-V characteristics show step like features. Step 1 with V_S1_ ≈ −70V was observed at temperatures above 200 K and step 2 with V_S2_ ≈ −1V occurred at temperatures above 350 K. The steps in the I-V characteristics normally correspond to the ultimate filling of trap centers by injected carriers [15,16]. The voltage V_s_ at which the step onsets, is given by,

(1) $$V_{s}=\frac{{\mathrm{qL}}^{2}}{2\varepsilon_{\mathrm{SiC}}}\times p_{t,0}$$

The concentration of trap centers with activation energy $E_{t}$, unoccupied by the injected carriers, ($p_{t,0}$) is given by $\frac{N_{t}}{g}\exp\left[\frac{E_{t}-F_{0}}{k_{B}T}\right]$. Here, $k_{B}$ is the Boltzmann’s constant, L is the thickness of the epitaxial layer, q is the electronic charge, $\varepsilon_{\mathrm{SiC}}$ is the permittivity of the material, $F_{0}$ is the Fermi energy, $N_{t}$ is the trap concentration, and g is the degeneracy factor. Using Equation (1) for the step voltages V_S1_ = −70 V and V_S2_ = −1 V, the trap densities for the centers unoccupied by the injection carriers was calculated to be $2\times 10^{14}$ cm^−3^ and $3\times 10^{12}$ cm^−3^, respectively. The former trap concentration was assumed to be the total trap concentration considering traps corresponding to step 1 were emptied at V=0.

**Figure 3:**
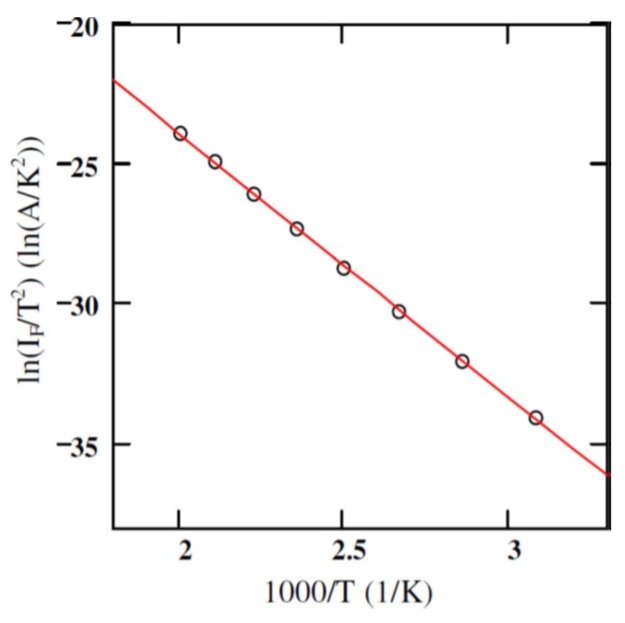
Activation energy plot for a 50 µm n-type 4H-SiC epitaxial layer (n-M50) SBD with 3.2 mm diameter Ni contact. The solid red line shows the linear fit to the experimental data.

From a similar study on a n-type (n-M50) sample, the variation of barrier height as a function of temperature was investigated [17]. It was observed that Ni on 4H-SiC epitaxial layer formed a typical Schottky junction. Unlike the n-SI50 samples, no step like features were noticed in the reverse bias characteristics of these samples. Using the activation energy method described in [16], the electrically active area A and the effective Richardson constant $A^{**}$ was calculated. The expression of a forward current $I_{F}$ as a function of forward bias voltage $V_{F}$, in a Schottky junction can be expressed as in Equation (2).

(2) $$\ln\left(\frac{I_{F}}{T^{2}}\right)=\ln\left({\mathrm{AA}}^{**}\right)-q\left(\phi_{B}-V_{F}\right)/k_{B}T$$

where, *T* is the absolute temperature, q is the electronic charge, and $\phi_{B}$ is the Schottky barrier height. Figure 3 shows the plot of $\frac{I_{F}}{T^{2}}$ as a function of inverse of absolute temperature (1000/T). A linear fit to the curve gives the the activation energy $q\left(\phi_{B}-V_{F}\right)$ and the product ${\mathrm{AA}}^{**}$ from the slope and intercepts, respectively. The studies were conducted in the temperature range 325–500 K with $V_{F}=0.8\;V$. Using a geometrical area of 0.08 cm^2^, the effective Richardson constant was found to be 0.074 Amp/K^2^.cm^2^ which is three orders of magnitude lower than the theoretically predicted value of 146 Amp/K^2^.cm^2^. Moreover, if the theoretical value of $A^{**}$ is considered then the effective geometrical area of the detector turns out to be 0.008 cm^2^ which is an order of magnitude less than the actual geometrical area. The reason behind obtaining a lower value of $A^{**}$ is thought to be due to using the geometrical area in the calculations, instead of the effective electrically active area. It is a well known fact that the measured currents in an I-V measurement correspond to the currents flowing through the patches with lower surface barrier height only [18,19]. 

The effect can be more pronounced in Schottky contacts with large spatial variation of barrier heights. As a result, the resulting effective area through which any current could flow is lower than the actual geometric contact area.

**Figure 4:**
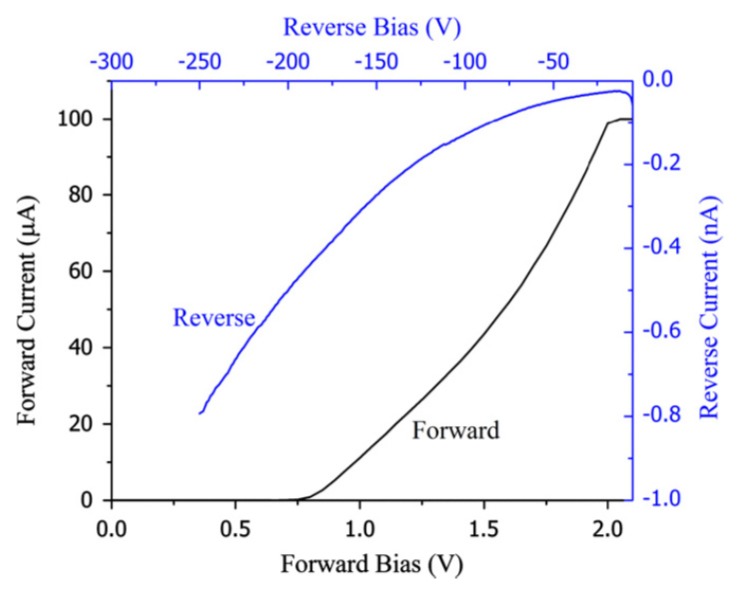
Forward and reverse I-V characteristics obtained for 50 µm n-type 4H-SiC epitaxial layer (n-S50) SBD with 3.8 mm diameter Ni contact.

Figure 4 shows the I-V characteristics obtained for a 50 µm n-type Ni/4H-SiC (n-S50) epitaxial layer detector [20]. Like the n-M50 samples, these samples also showed clearly the formation of Schottky type junction. Currents as low as 0.2 nA for −100 V was obtained and the Schottky junction was observed to be conducting for bias voltages below +1.0 V. Hence a positive bias on the Ni/n-4H-SiC interface constitutes a forward biased Schottky junction. These reverse biased I-V curves, in contrast to the SI epitaxial layers, did not exhibit any step like regions either, indicating the low concentration of those trap centers responsible for the step like structures in the SI samples. The diode ideality factor and Schottky barrier height were calculated from the forward I-V characteristics using a thermionic emission model [19]. According to the model, the current (I) for an asymmetric junction (Schottky in this case) is related to the applied bias $V_{a}$ across the junction, in the following manner

(3) $$\ln\left(I\right)\sim \left(\frac{\beta }{n}\right)\ln\left(V_{a}\right)+\ln\left(I_{s}\right)$$

where, $I_{s}$ is the saturation current, n is the diode ideality factor, and $\beta =q/k_{B}T$*,* q being the electronic charge and T the absolute temperature. The saturation current, according to the model, is given by

(4) $$I_{s}=A^{**}{\mathrm{AT}}^{2}\exp\left(-\beta \phi_{B}\right)$$

where, $A^{**}$ is the effective Richardson constant (146 Acm^−2^K^−2^ for 4H-SiC), A is the area of the diode, $\phi_{B}$ is the Schottky barrier potential. Hence, a semi-logarithmic plot of the diode current as a function of the bias voltage results in a straight line. As can be seen from Equation (3), the slope of the straight line can be used to calculate the ideality factor and the intercepts give the reverse saturation current. The diode ideality factor and the barrier height thus calculated were 1.4 and 1.3 eV, respectively. It can be noted that the barrier height calculated in the above fashion depends on the spatial homogeneity of the barrier heights. The locations with lower barrier heights will allow more currents compared to the regions with higher barrier heights [19]. The departure from the theoretical diode ideality factor of unity suggests that these Schottky contacts do have spatial non-uniformity of surface barrier height. 

**Figure 5:**
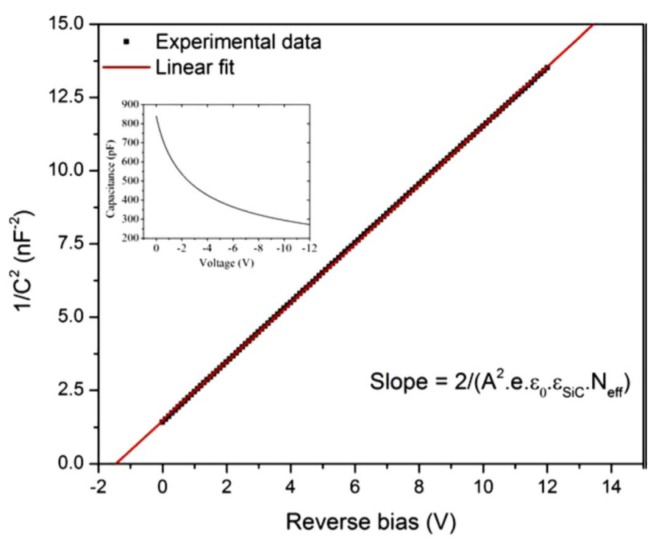
Plot of 1/C^2^ as a function of reverse bias voltage recorded at room temperature for a 50 µm n-type Ni/4H-SiC (n-S50) SBD. The original C-V plot is shown in the inset.

The SI 4H-SiC layers (SI-M50) showed a very low capacitance of ≈2 pF which hardly varied with applied bias, confirming its semi-insulating (SI) nature. The n-type epitaxial layers on the other hand showed high sensitivity of depletion capacitance with any change in the applied reverse bias. Figure 5 shows the variation of junction capacitance (C) as a function of the reverse bias voltage for the n-type 4H-SiC epitaxial (n-S50) samples. A straight line fit to the plot of $1/C^{2}$ as a function of reverse bias voltage gives the built-in potential and the effective doping concentration. The surface barrier height can also be calculated from the C-V measurements using the equations below.

(5) $$\phi_{B\left(C\text{-}V\right)}=V_{\mathrm{bi}}+V_{n}$$

where $V_{n}$ is the potential difference between the Fermi level energy and the bottom of the conduction band in the neutral region of the semiconductor and is given by

(6) $$V_{n}=k_{B}\mathrm{Tln}\frac{N_{C}}{N_{D}}$$

where $N_{C}$ is the effective density of states in the conduction band of 4H-SiC and is taken equal to $1.6\times 10^{19}$ cm^−3^ [15]. The effective doping concentration and the built-in potential was calculated to be $1.1\times 10^{15}$ cm^−3^ and 1.4 V, respectively. The $\phi_{B\left(C\text{-}V\right)}$ was calculated to be 1.47 eV using Equations (5) and (6) which is slightly higher compared to that calculated from the I-V measurements (1.3 eV). The difference can be attributed to the difference in the underlying mechanism of the two measurements. As has been mentioned earlier, the current flow in the I-V measurements is mostly in the low barrier height regions and hence the barrier height reflects those regions only, whereas the C-V measurements gives the average value of the barrier height for the entire surface area of the Schottky contact. More work is needed to further understand the defects in the material and devise preparation approaches such as surface passivation and edge termination for mitigating these effects [21,22].

### 3.2. Quality Evaluation of Epitaxial Layers

**Figure 6:**
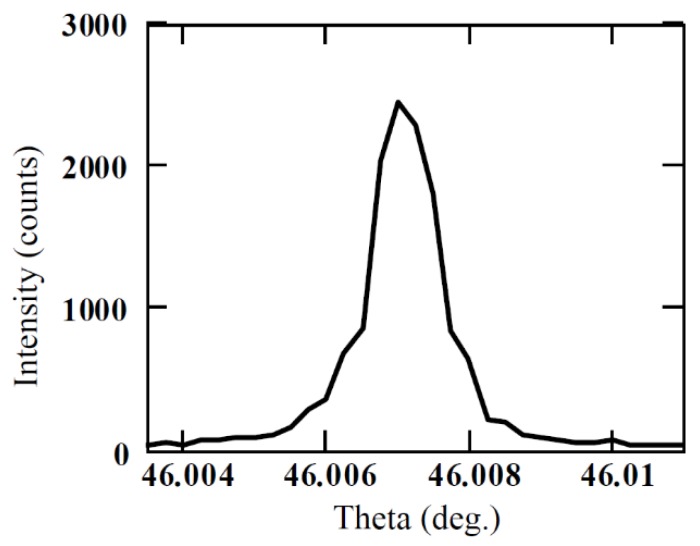
Rocking curve ((0008) reflection) of the 4H-SiC semi-insulating epitaxial layer (SI-M50) used for detector fabrication.

The performance of a device obviously depends on the single crystallinity of the epitaxial layer. XRD rocking curve measurement gives very accurate information on the orientation of crystallographic planes. Preferential or defect delineating etching helps in exposing the sites around defects like dislocations, stacking faults, precipitates, and point defects. The width (FWHM) of the XRD rocking curve peak is a measure of the crystalline quality. The lower the FWHM, the higher the crystalline quality [23]. Molten KOH is well-known for its preferential etching on the SiC surface at defect sites [24]. Quality of the semi-insulating 4H-SiC epitaxial layers, used for detector fabrication in this work, was assessed using defect delineating chemical etching in molten KOH followed by XRD rocking curve measurements at the exposed defect sites. For the reflection geometry used in our studies, FWHM of the rocking curve can be calculated [24,25] using the following equation

(7) $$\mathrm{FWHM}=\frac{2r_{e}\lambda^{2}}{\pi V\sin2\theta_{B}}\frac{1}{\sqrt{\gamma }}\left|C\right|\sqrt{F_{\mathrm{hkl}}F_{\bar{h}\;\bar{k}\;\bar{l}}}$$

where $r_{e}$ is the classical electron radius, λ is the X-ray wavelength, V is the volume of the unit cell, $\theta_{B}$ is the Bragg angle, $\gamma =\cos\left(\psi_{h}\right)/\cos\left(\psi_{0}\right)$ is the asymmetric ratio, where $\psi_{h}$ and $\psi_{0}$ are the angles between the normal to the crystal surface directed inside the crystal and the reflected and incident directions of X-ray waves, respectively, C is the polarization factor (C=1 for σ polarization and $C=\cos2\theta_{B}$ for π polarization), and $F_{\mathrm{hkl}}$ is the structure factor with the modulus for (000l) reflection in 4H-SiC (back-reflection geometry) given by equation below [24].

(8) $$\left|F_{000l}\right|=4\sqrt{f_{\mathrm{Si}}^{2}+f_{C}^{2}+2f_{\mathrm{Si}}f_{C}\cos\left(3\pi l/8\right)}$$

where $f_{C}$ and $f_{\mathrm{Si}}$ are the atomic scattering factors of C and Si atoms, respectively. The scattering factors were calculated using the nine-parameter equation given below [25,26,27].

(9) $$f=c+\overset{4}{\underset{i=1}{\sum }}a_{i}\exp\left[-b_{i}\sin^{2}\left(\theta /\lambda \right)\right]$$

where $a_{i}$, $b_{i}$, and c are the atom-specific Cromer–Mann coefficients, which can be found in [26,27,28]. The FWHM of the (0008) plane reflection was calculated using Equations (7)–(9) and was found to be less than 2.7 arc sec. Figure 6 shows the experimentally obtained rocking curve for (0008) reflection for a semi-insulating 50 µm thick 4H-SiC epitaxial layer [17]. The FWHM of the rocking curve peak was found to be ≈3.6 arc sec, revealing high quality of our epitaxial layer. Structural defect densities were also estimated using a Nomarski microscope. An etch pit density (EPD) of threading screw dislocations (TSDs) was found to be ≈1.7×10^3^ cm^−2^. The concentration of the threading edge dislocations (TEDs) was calculated to be ≈1 × 10^4^ cm^−2^ and basal plane dislocation (BPD) density was found to be ≈70 cm^−2^.

### 3.3. Radiation Detection Measurements 

**Figure 7:**
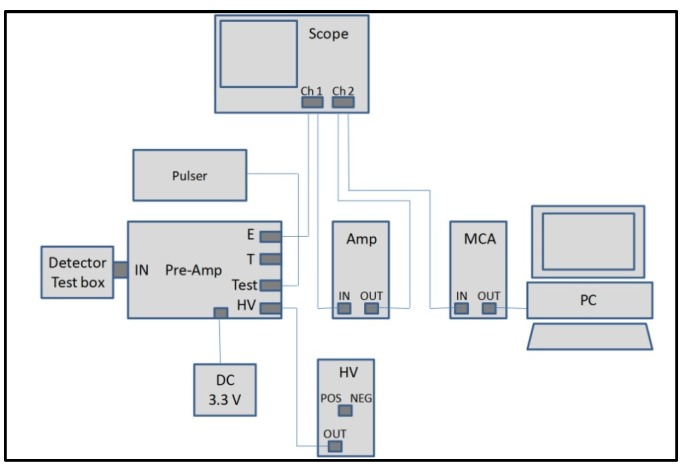
Schematic of the radiation detection set-up.

Most of the following radiation detection measurements were undertaken in the spectrometer configuration shown in Figure 7. The details of the components are described in Section 2.

#### 3.3.1. Electron-Hole Pair Creation Energy Measurements

Electron-hole pair (ehp) creation energy, henceforth designated by ε, determines the energy resolution of a radiation detector as the energy resolution is directly linked to the number of electron-hole pairs created for a single incoming radiation event. The higher the number of ehp, the higher the resolution. A method of iterative determination of the ε value which involves an absolute calibration using a precision pulser to match the alpha peak energy (5486 keV) observed using a high-resolution 4H-SiC n-type epitaxial Schottky detector has been reported in our earlier publication [29]. The alpha particle spectrometer was calibrated electronically by injecting a pulser signal of known pulse height, $V_{\mathrm{pulser}}$ (mV), from a precision pulser through a calibrated feed-through capacitor $C_{\mathrm{test}}$, to the preamplifier input. The peak position of the shaped pulses was recorded in a multi-channel analyzer for a set of known pulse-height inputs. The SiC equivalent of the MCA peak positions, $E_{\mathrm{pulser}}$ in keV were calculated using the equation given below:

(10) $$E_{\mathrm{pulser}}=\frac{V_{\mathrm{pulser}}\times \varepsilon \times C_{\mathrm{test}}}{q}$$

where q is the electronic charge. A linear regression of the SiC equivalent peak position as a function of MCA channel number was used to calculate the calibration parameters. The detectors used for this study were fabricated on a 20 µm n-type 4H-SiC epilayer. The ε value we obtained using the given procedure was 7.28 eV. The value thus calculated differs from the widely accepted value of 7.7 eV as reported earlier [30]. Rogalla et al. calculated an ε value of 8.4 eV for alpha particles in semi-insulating 4H-SiC [31]. An ε value of 8.6 eV for alpha particles in epitaxial n-type 4H-SiC were reported by Lebedev et al. [32]. Ivanov et al. [33] have reported ε = 7.71 eV for alpha particles in epitaxial n-type 4H-SiC. An ε value of 7.8 eV has been reported by Bertuccio and Casiraghi for 59.5 keV gamma rays [34].

#### 3.3.2. Minority Carrier Diffusion Length Measurements

**Figure 8:**
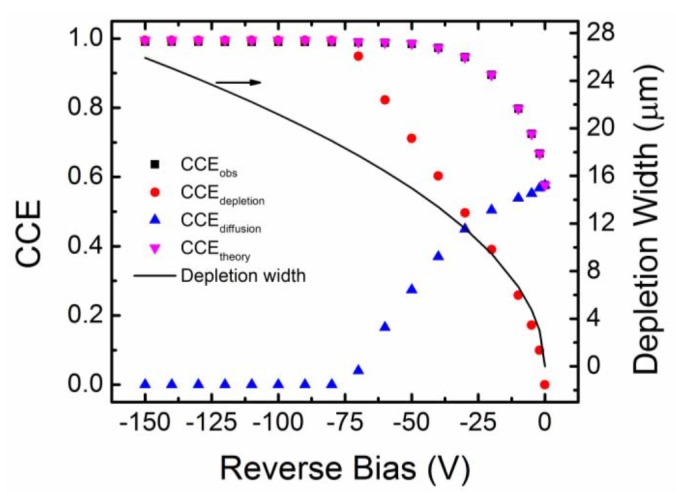
Variation of $CCE_{obs}$ and $CCE_{theory}$ with reverse bias voltage for an n-type Ni/4H-SiC (n-S20) SBD. The contributions of $CCE_{depletion}$ and $CCE_{diffusion}$ to the total $CCE_{obs}$ are also plotted. The solid line shows the variation of depletion width with bias.

Minority carrier diffusion length is the average distance a minority carrier traverses before it recombines. Higher minority carrier diffusion length obviously enhances the detection properties by reducing the effect of ballistic deficit [35]. The minority carrier diffusion length can be indirectly calculated by observing the variation of charge collection efficiency of detectors for ionizing radiations like alpha particles with reverse bias voltage. Charge collection efficiency (CCE) is defined as the ratio of charge collected by the collecting electrode at a particular bias to the maximum collected charge, assuming all the charge carriers have been received by the collecting electrode. The collected charge is generally calculated by integrating the current signal received at the input of a charge-sensitive pre-amplifier. Alternatively, the CCE can also be calculated from alpha spectroscopic measurements. The MCA peak position $E_{p}$ due to a monoenergetic alpha source can be predicted in a properly calibrated alpha spectrometer, assuming that all the charge carriers created by that particular energy have been received by the collecting electrode. The CCE can then be determined for any MCA peak at $E_{\alpha }$ by calculating the ratio $E_{\alpha }/E_{p}$. Figure 8 shows the variation of observed charge collection efficiency ($CCE_{obs}$) of a n-type Ni/4H-SiC SBD as a function of reverse operating bias voltage [36,37]. By noting that the movement of the charge carriers in rectifying junctions can be due to diffusion as well as drifting, the contribution of each mechanism to CCE can be calculated using a proper model. The contribution of the drift $CCE_{depletion}$ and diffusion $CCE_{diffusion}$ related charge collection to the $CCE_{obs}$ was calculated using a drift-diffusion model [38] summarized below in Equation (11).

(11) $${\mathrm{CCE}}_{\mathrm{theory}}=\frac{1}{E_{p}}\int_{0}^{d}\left(\frac{\mathrm{dE}}{\mathrm{dx}}\right)\mathrm{dx}+\frac{1}{E_{p}}\int_{d}^{x_{r}}\left[\left(\frac{\mathrm{dE}}{\mathrm{dx}}\right)\times \exp\left\{-\frac{\left(x-d\right)}{L_{d}}\right\}\right]\mathrm{dx}$$

$$={\mathrm{CCE}}_{\mathrm{depletion}}+{\mathrm{CCE}}_{\mathrm{diffusion}}$$

where d is the depletion width at the particular bias, $\frac{dE}{dx}$ is the electronic stopping power of the alpha particles calculated using SRIM [39], $x_{r}$ is the projected range of the alpha particles with energy $E_{p}$. We fitted the $CCE_{theory}$ values to the $CCE_{obs}$ values considering $L_{d}$, the minority carrier diffusion length, as a free parameter. The $L_{d}$ value corresponding to the best fit was returned as the average minority carrier diffusion length. For the present SBD, the average value of $L_{d}$ was found to be ≈18.6 µm. From Figure 8 it was also observed that the $CCE_{diffusion}$ values dominate considerably over that of $CCE_{depletion}$ up to a reverse bias of −30 V. At even higher bias voltages the depletion width becomes equal or more than the projected range of alpha particles (≈18 µm in SiC for 5486 keV alpha particle) and hence charge collection was mainly due to the drifting of charge carriers within the depletion width. Hence, above bias voltage of −70 V, the $CCE_{depletion}$ matched the $CCE_{obs}$ values. 

#### 3.3.3. Alpha Particle Pulse-Height Spectroscopy (PHS)

**Figure 9:**
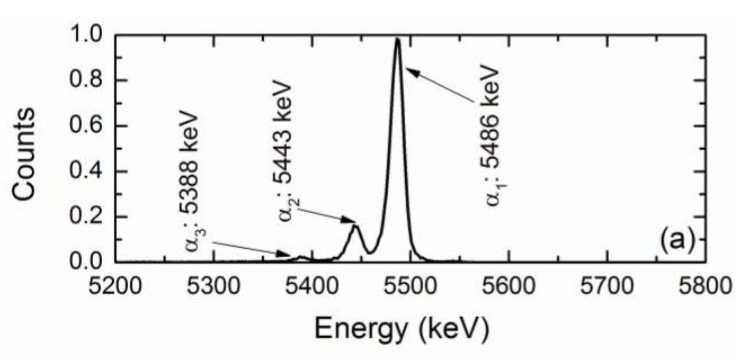
A pulse-height spectrum obtained for a 20 µm n-type 4H-SiC (n-S20) SBD and ^241^Am alpha source.

The n-type 4H-SiC epitaxial layer detectors were put to test as an alpha particle detector for precise energy measurements. A ^241^Am alpha particle source was used as the test source. Figure 9 shows a typical pulse-height spectrum obtained from a n-type 4H-SiC SBD [40]. As is evident from the Figure 9, the detector clearly resolved the three primary alpha particles emitted by a ^241^Am alpha source. The percentage energy resolution (in terms of full width at half maxima or FWHM) for the 5486 keV line was calculated to be 0.29% with a ≈100% charge collection efficiency. The observed alpha energy resolution is so far the best-reported value to date in the literature. At this point, it is also worth discussing the evolution of 4H-SiC epitaxial layers as alpha particle detectors. The idea to use 4H-SiC was pioneered by Babcock and Chang [1]. Ruddy et al. [41] reported a percentage energy resolution of 5.8% and 6.6% for a deposited energy of 294 and 260 keV alpha particles, respectively. It can be noted that the authors used a collimated ^238^Pu source and circular diode contacts of 200 and 400 µm. Later on, Ruddy et al. also reported [42] an energy resolution of 5.7% for a deposited energy of 89.5 keV alpha particles from a 100 µm collimated ^148^Gd source in similar detectors with comparatively larger Schottky contact diameter of 2.5, 3.5, 4.5, 6.0, and 10 mm thick 4H-SiC epitaxial layers. In another work [8], Ruddy et al. reported an energy resolution close to 46 keV (≈0.8%) for alpha particles from a ^238^Pu source and 41.5 keV (≈1.3%) for alpha particles from a ^148^Gd source for devices with an aluminum guard ring. Ivanov et al. [43] reported an energy resolution of 20 keV (≈0.4%) in the energy range 5.4–5.5 MeV. In yet another work, Ruddy et al. [44] reported an energy resolution of 20.6 keV (≈0.4%) for ^238^Pu alpha particles. In our earlier work [20] we reported an energy resolution of 2.7% for 5486 keV alpha particles in 50 µm thick n-type Ni/4H-SiC detectors. The energy resolution mentioned in the above works were found to be primarily dependent on the defect type and concentrations within the 4H-SiC epilayers. The nature of defects, which controls the detector properties, will be described in detail in a later section. 

#### 3.3.4. Low Energy Gamma Spectroscopy

**Figure 10:**
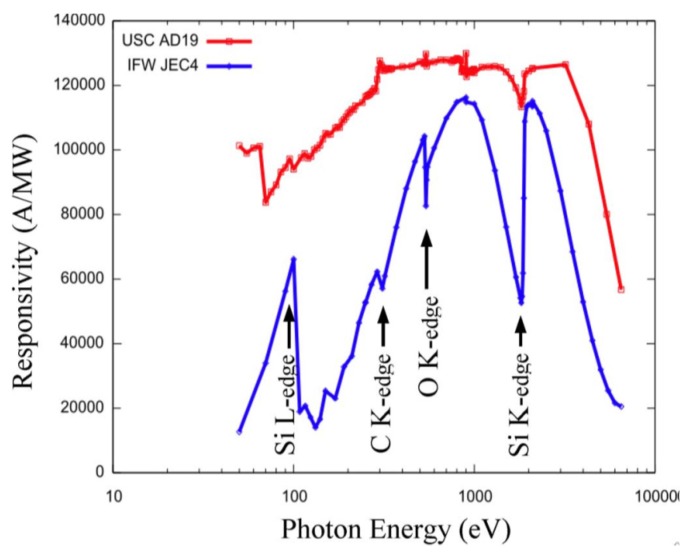
Measured responsivity of a 4H-SiC n-type epitaxial device biased to 250 V and an IFW JEC4 photodiode biased to 120 V.

Absolute measurement of photo-responsivity and probing of physical construction of photonic sensors can be very effectively done using synchrotron light sources. N-type 4H-SiC epitaxial layer detectors fabricated at UofSC were studied at NSLS at BNL for detection of low energy X-rays. The results were compared to a commercial off-the-shelf (COTS) SiC UV photodiode by IFW optronics GmbH (Jena, Germany), model JEC4 which was known to be the best commercially available for such applications [45]. An X-ray spectrometer for such a low energy spectral range is not available commercially. Figure 10 shows the responsivity of one of our detectors and a IFW JEC4 SiC UV photodiode to soft X-ray energy ranges biased at 250 and 120 V, respectively [17]. The following results were derived using a statistical analysis of these data based on energy-dependent X-ray attenuation lengths [46].

**Figure 11:**
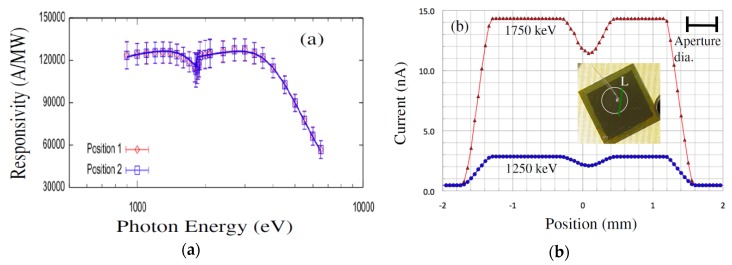
(**a**) Responsivity of the detectors on 4H-SiC n-type epitaxial layer at two different locations and (**b**) surface scan profiles along line L obtained to assess the detector’s uniformity.

Responsivity measurements were carried out using the U3C [45] and X8A [46] lines by recording successive measures of photocurrent in response to a high flux, mono-energetic beam of photons in a well-calibrated silicon sensor (with known responsivity) and in the sensor of interest (Figure 11a). Dead layers and a limited active volume thickness led to responsivity that varies greatly with photon energy. Further, edges were also apparent in the responsivity curve, arising from discrete atomic transitions. Edges associated with silicon and carbon is clearly observed in the data, providing a quantitative measure of the composition and dimension of the active and dead layers [46]. The general feature of a steep decrease starting at 2–3 keV provides information on active layer thicknesses, which is deduced to be 21 µm in our detector compared to roughly 6 µm for the JEC4 diode [47]. Due to the higher active layer thickness our sensor chip showed significant improvement of responsivity in the few keV range compared to COTS SiC UV photodiode. Our detector has shown much higher response in the low energy part of the spectra as well, which could be attributed to a much thinner dead/blocking layer, deduced from the responsivity curve to result solely from the 10 nm thick nickel layer (which leads to the pronounced edge at 70 eV). In comparison, the JEC4 diode has been found to include a significant oxidation and inactive SiC layer on the order of 100 nm each, which limits responsivity at low photon energies [47]. It should be noted that the JEC4 diode is intended for UV detection, for which it is well suited. The significant dead layers are likely due to passivation, which may be preferred over reducing the thickness of dead layers on the active face of the sensor.

**Figure 12:**
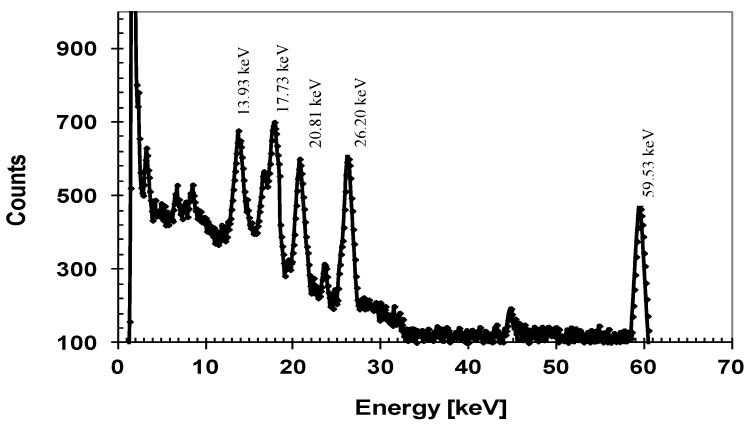
^241^Am spectrum of a 4H-SiC detector (8.0 mm^2^) at 300 K, 250 V bias, and 4 μs shaping time.

Our detectors also exhibited very good spatial uniformity in measured responsivity. Figure 11b shows responsivity at two different locations and line scan profiles for two different X-ray energies. Note that the decrease of the detector’s current at about 0 mm (Figure 11b) is due to the crossing of the location of wire bonding and not due to the detector’s imperfection. The SiC detectors were connected to low noise front end electronics developed in-house for pulse-height spectroscopy (PHS). Pulse height spectra measurements showed high resolution of our 4H-SiC detectors in detecting 59.6 keV gamma rays from ^241^Am. Figure 12 shows a pulse height spectrum recorded using an ^241^Am radiation source with the detector biased at 250 V, with an FWHM of 1.2 keV (2.1%) at 59.5 keV, which is comparable to the resolution achieved using high quality CdZnTe detectors [48,49].

### 3.4. Electronic Noise Measurements

The energy resolution of nuclear energy spectrometers is dependent on the noise of the detector and associated electronic modules in the spectrometer and the pre-amplifier in particular. Noise is defined as any statistical fluctuation in currents measured in the detector or associated electronics which constitute a signal. The most appropriate way to monitor the noise is to capture the pulses from a standard pulser along with the pulses produced by a detector due to the incoming radiation. The pulse-height spectrum then reveals a peak due to the pulser with the actual radiation peaks. The width (FWHM) of the pulser peak then gives the idea of the overall noise of the spectrometer ($FWHM_{total}$). 

**Figure 13:**
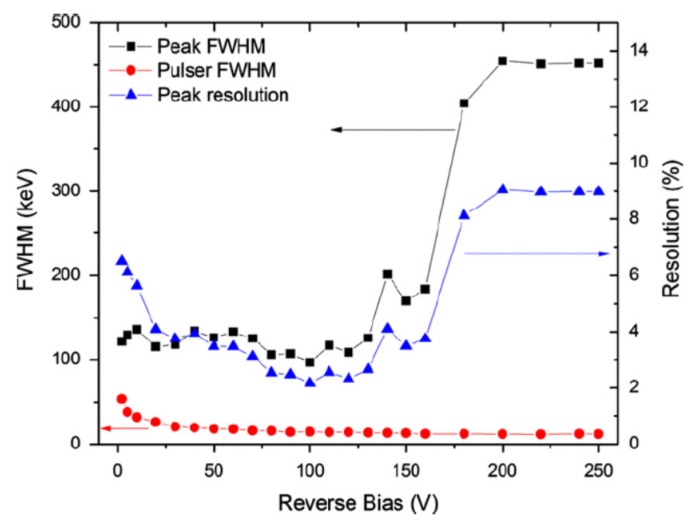
Variation of 5.48 MeV alpha peak FWHM, pulser peak FWHM, and alpha peak percentage resolution as a function of detector bias voltage.

Figure 13 shows the results of a typical noise monitoring measurement [36,37]. The energy resolution of the detector could be seen to improve with increase in bias voltage up to 100 V reverse bias because of the increase in depletion width (active volume of the detector) and lowering in capacitance. The energy resolution beyond 100 V was seen to deteriorate with increase in bias. The increase in leakage current was ruled out as an explanation as it could be seen from the figure that the pulser noise did not increase at all. A possible reason behind the deterioration of the resolution is incorporation of the threading dislocation as the depletion width approaches towards the epilayer–substrate interface with the increase in reverse bias. The epilayer–substrate interfacial region is prone to have a larger threading type dislocation concentration, which propagates from the substrate to the epilayer [36].

For a superior quality n-S20 detector, the ${\mathrm{FWHM}}_{\mathrm{total}}$ was found to be 19.8 keV for 5486 keV alpha particles. The contribution from the noise from the front-end electronics (${\mathrm{FWHM}}_{\mathrm{elec}}$), and the detector leakage current (${\mathrm{FWHM}}_{\mathrm{leakage}}$), can be found from the width of the MCA pulser peak recorded with the detector plugged in and biased. The collective broadening due to ${\mathrm{FWHM}}_{\mathrm{elec}}$ and ${\mathrm{FWHM}}_{\mathrm{leakage}}$ for this detector was found to be 15.9 keV. The other contributions to the ${\mathrm{FWHM}}_{\mathrm{total}}$ are from the statistical fluctuation in the number of ehps produced by ionizing radiation ${\mathrm{FWHM}}_{\mathrm{stat}}$, and broadening due to variation of energy due to the entrance window, the angle of incidence, self-absorption in the source, etc. $\left({\mathrm{FWHM}}_{\mathrm{other}}\right)$. All these factors along with the intrinsic detector resolution ${\mathrm{FWHM}}_{\det}$ are related to the ultimate peak broadening through the relation given in Equation (12). ${\mathrm{FWHM}}_{\mathrm{stat}}$ and ${\mathrm{FWHM}}_{\mathrm{other}}$ were calculated in this detector and found to be 5.3 and 0.44 keV, respectively [29]. The intrinsic detector resolution was calculated from Equation (12) and found to be 10.5 keV.

(12) $${\mathrm{FWHM}}_{\mathrm{total}}{}^{2}={\mathrm{FWHM}}_{\det}{}^{2}+{\mathrm{FWHM}}_{\mathrm{leakage}}{}^{2}+{\mathrm{FWHM}}_{\mathrm{stat}}{}^{2}+{\mathrm{FWHM}}_{\mathrm{elec}}{}^{2}+{\mathrm{FWHM}}_{\mathrm{other}}{}^{2}$$

The electronic noise has various sources such as detector leakage current and capacitance, and input FET (field effect transistor in the pre-amplifier) noise. The contribution of the different sources to the signal-to-noise ratio is dependent on the filtering or shaping operation and in particular the shaping time (except the FET noise). A clear understanding of the electronic noise is thus very essential to proper tuning of the spectrometer and a pioneering work in this area has been conducted by Bertuccio and Pullia [50]. The electronic noise was expressed in terms of equivalent noise charge (ENC) and plotted as a function of the shaping time τ of the shaping amplifier. The plots were then fitted to Equation (13) below using a least square estimation method.

(13) ENC2=(aCtot2A1)1τ+[(2πafCtot2+bf2π)A2]+(bA3)τ

where $C_{\mathrm{tot}}$ is the total input capacitance, $A_{1}$*,* $A_{2,}$ and $A_{3}$ are constants depending on the shaping network response, a is the coefficient of white series noise contribution due to the thermal noise of the FET channel, $a_{f}$ is the coefficient of the FET 1/f noise, $b_{f}$ is the dielectric noise coefficient, and b is the coefficient related to the sum of the white parallel noise due to the shot noise and the detector leakage current.

**Figure 14:**
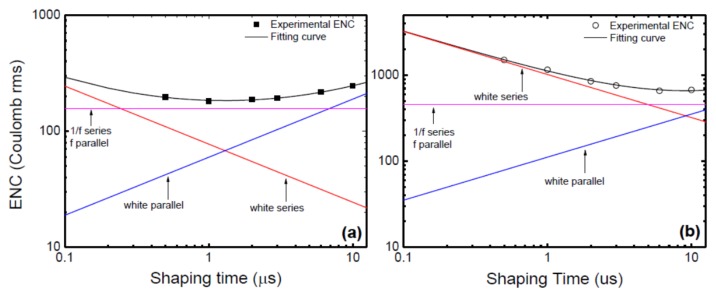
Variation of equivalent noise charge as a function of shaping time constant (**a**) without the detector connected, (**b**) with the detector connected.

Figure 14a,b shows the variation of ENC with shaping time without and with the detector (20 µm n-type Ni/4H-SiC (n-S20) SBD) connected [37]. The contributions from the three different terms were plotted separately. The minimum noise without the detector was observed to correspond to a shaping time value between 1 and 2 µs. The same shifted to a higher range of shaping time (between 3 and 6 µs) when the detector was plugged in. It can also be seen that the white parallel noise increased almost by a factor of five after connecting the detector and the pink noise marginally increased for any given τ after connecting the detector. In contrast, the white series noise increased by an order of magnitude when the detector was connected. The increase in white parallel noise can be attributed to the increase in the leakage current (from the detector) and the increase in white series and parallel noise is supposedly due to the increase in input capacitance when the detector is plugged in.

### 3.5. Defect Level Characterization

#### 3.5.1. Morphological Defect Study using Electron Beam Induced Current (EBIC) Studies

**Figure 15:**
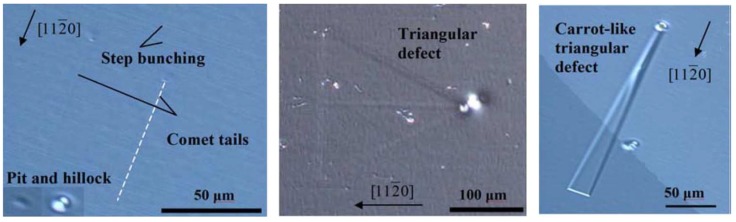
Microscopic image of the morphological defects revealed after KOH etching in n-type 4H-SiC samples.

**Figure 16:**
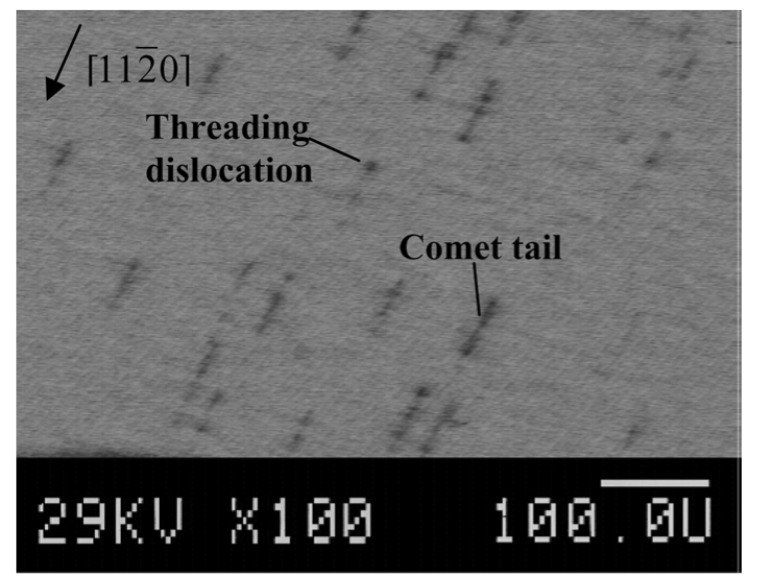
Electron beam induced current spectroscopy (EBIC) image of a n-type 4H-SiC epitaxial layer.

The SI (SI-M50) and n-type 4H-SiC (n-M50) epitaxial layer samples have been studied for the presence of morphological defects [51]. Figure 15 shows the morphological defects that were observed in the n-type epitaxial layer samples followed by KOH etching, optical microscopy, and EBIC studies [52]. While the n-type epitaxial layers showed features like comet tails, pits, hillocks, triangular defects, and step bunching, the SI epitaxial layers showed the presence of carrot defects only. The hillocks were proposed to originate from foreign impurities and silicon precipitates. Silicon can also have pits if it evaporates during the epitaxial growth. Triangular defects indicate the inclusion of 3C-SiC and Shockley-type stacking faults nucleating on micropipes and elementary threading screw and edge dislocation [53]. The presence of these defects is believed to increase the leakage current upon application of high electrical fields to the devices [54]. Pits and step bunching types of morphological defects are not believed to influence the leakage current but can interfere with proper functioning of the Schottky contacts in case the epilayer surface is not smooth enough due to their presence [54]. Figure 16 shows an EBIC image of an n-type 4H-SiC epitaxial layer. The typical signatures of threading dislocation type defects could be seen as black spots [55,56]. The EBIC features were mapped on to the morphological defect images and it was correlated that the dark spots are the signatures of the comet tail morphological defects in the n-type epitaxial layers. The superior samples did not show any presence of morphological defects.

#### 3.5.2. Thermally Stimulated Current (TSC) Measurements

**Figure 17:**
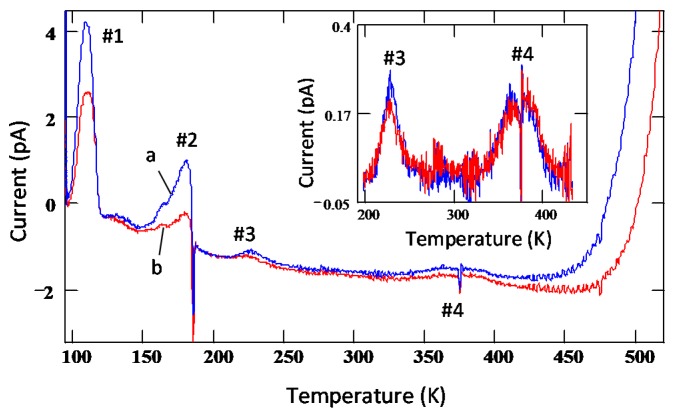
Thermally stimulated current (TSC) spectra obtained using an n-type 4H-SiC (n-M50) epitaxial layer at two different reverse bias voltages: (**a**) 12 V and (**b**) 4 V.

Thermally stimulated current measurement is yet another sensitive technique to study defects in semi-insulating (SI) as well as conducting samples. Figure 17 shows TSC spectra obtained from an n-type 4H-SiC epitaxial layer reverse biased at two different voltages, 4 and 12 V [56]. The spectra were acquired with a heating rate of 15 K/min. Four discernable peaks were observed in both the TSC spectra and were numbered 1 to 4. Peak#1 has the most prominent presence with maximum temperature T_m_ = 109 K and an activation energy ≈0.25 eV as estimated from the Arrhenius plot (not shown). This peak can be associated with shallow acceptor-like levels situated at 0.25 eV from the valence band edge and may be related to Al- and/or B-impurities as well as to their complexes with intrinsic defects [56]. It can be noticed that the intensity of the peak#1 showed voltage dependence, which implies that the deep level centers are distributed over a thickness of 4 μm in the epilayer as 4 μm is the depletion width achieved at 12 V. The concentration of the associated defects was estimated to be ≈7 × 10^13^ cm^−3^, assuming uniform distribution of deep level centers in the depletion region. Equation (14) was used to calculate the trap concentrations, $N_{T}$:

(14) $$N_{T}=\frac{Q}{A}\sqrt{\frac{2N_{d}}{q\varepsilon_{SiC}\varepsilon_{0}\left(V_{bi}+V_{a}\right)}}$$

where, *Q* is the total charge emitted by the given trap which in turn is determined by the area under the corresponding peak, *A* is the contact area, $N_{d}$ is the effective doping concentration, $V_{bi}$ is the built-in potential, $V_{a}$ is the applied bias voltage, *q* is the electronic charge, $\varepsilon_{SiC}$ is the dielectric permittivity of SiC, and $\varepsilon_{0}$ is the dielectric permittivity of vacuum. 

It can be noted here that being a shallow defect level, the trap center corresponding to peak #1 is not expected to cause significant trapping/polarization even though its concentration is relatively high. The intensity of peak#2 was also observed to increase with reverse bias voltage. However, it depended on other conditions such as pumping time. Overnight pumping, since the exposure of the TSC chamber and the sample to air, resulted in decrease of the intensity of peak#2 by a factor of two to three. This suggests that peak#2 could have contributions from levels/dipoles produced by adsorption of residues in the vacuum chamber (such as water) onto the surface of the sample. Additionally, peak#2 was always distorted by the negative spike, the origin of which could not be explained. The intensities of peaks #3 and #4 do not show voltage dependence of the peak intensities as can be observed when zoomed-in (inset in Figure 17). This is an indicative of the fact that the traps associated with peaks #3 and #4 are located mostly near the metal-semiconductor interface and not in the interior of the epitaxial layer. The activation energies for peaks #3 and #4 could not be determined as the TSC signals were too weak. However, the traps corresponding to peaks #3 and #4 could be identified using their maximum temperatures and previously reported data in similar samples [12,14,56]. The peak#3 (T_m_ ≈226 K) can be assigned to D-center, a boron (B) related defect, boron at C-site (B_C_) or boron at Si site (B_Si_), and carbon vacancy V_C_ [57], whereas peak#4 can be assigned to intrinsic defects such as IL2 center [58,59].

**Figure 18:**
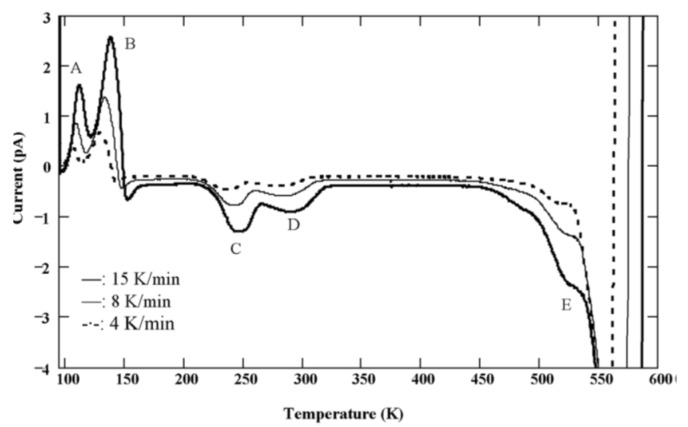
TSC spectra obtained using a SI 4H-SiC (SI-M50) epitaxial layer for three different heating rates.

**Figure 19:**
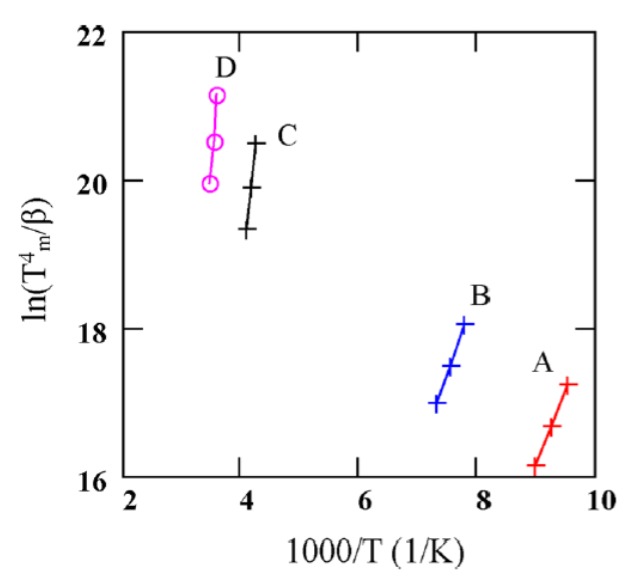
Arrhenius plots obtained from TSC spectra of a SI 4H-SiC (SI-M50) epitaxial layer at 0V bias. voltage.

Figure 18 shows TSC spectra obtained for a semi-insulating 4H-SiC epitaxial layer (SI-M50) sample at 0 V applied bias (higher bias resulted in large leakage currents for these samples) [14]. The thermally stimulated current at 0 V is due to a thermoelectric effect caused by small temperature gradient between the front and the back surface of the samples [60]. Five peaks of either polarity were observed and named as A–E. The positive peaks represent hole traps and the negative peaks represent electron traps as the polarity of the thermoelectric effect current is sensitive to the type of carrier. The Arrhenius plots of the designated traps, except for E, are shown in Figure 19. The trap parameters, calculated from the Arrhenius plots, and their identity are tabulated in Table 2. Due to high stray currents at high temperatures, peak E could not be used for Arrhenius plots. 

#### 3.5.3. Deep Level Transient Spectroscopy (DLTS) Measurements

**Figure 20:**
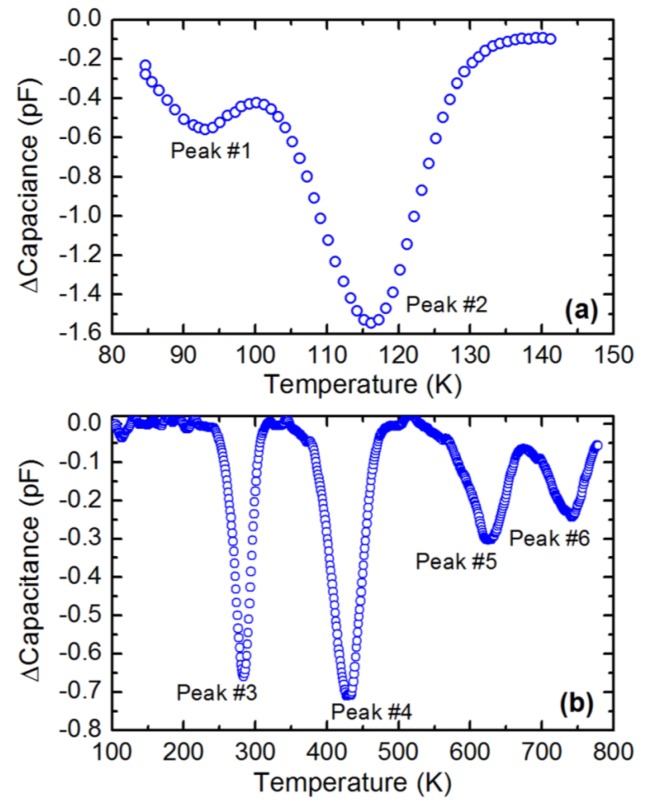
A typical deep level transient spectroscopy (DLTS) spectra obtained using the 50 µm n-type Ni/4H-SiC (n-S50) epitaxial Schottky barrier detector in a temperature range of (**a**) 80–140 K and (**b**) 80–800 K.

**Figure 21:**
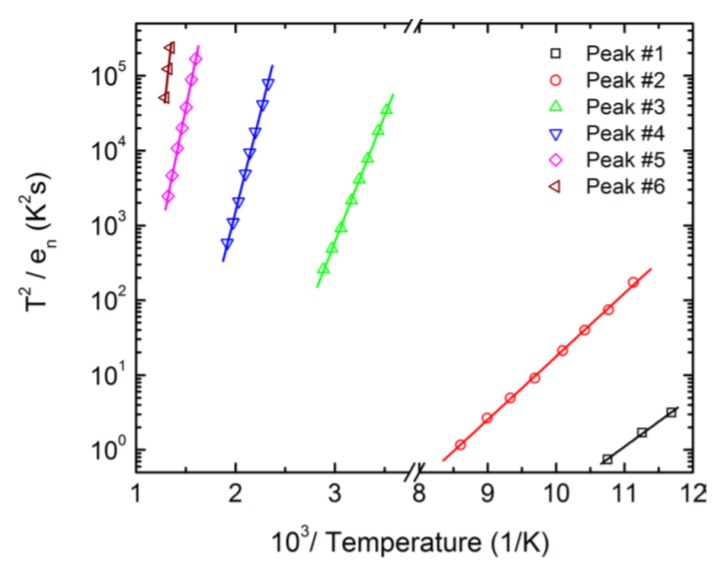
Arrhenius plots obtained for peaks #1–#6 corresponding to the DLTS spectra shown in Figure 20.

Deep level capacitance transient spectroscopy is a very sensitive technique to study deep level defects in semiconducting Schottky or p-n junction devices. Figure 20 shows a DLTS spectra obtained for a 50 µm thick n-type Ni/4H-SiC (n-S50) epitaxial Schottky barrier detector in the temperature range 85–790 K [63]. Six well-resolved peaks were observed. All the peaks, except the deepest one (peak#6), were identified using the existing literature. The Arrhenius plots are shown in Figure 21 and the extracted trap parameters are tabulated in Table 3. 

**Figure 22:**
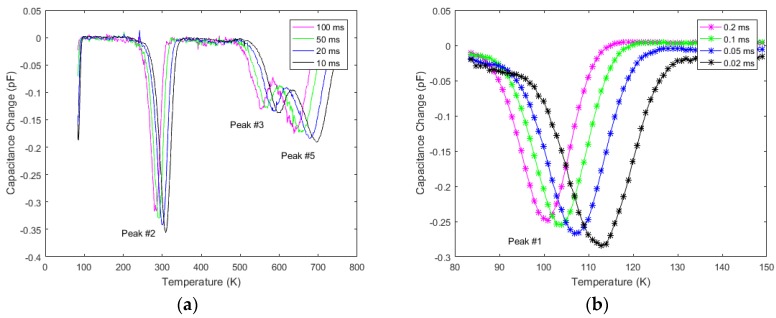
DLTS spectra of the detector obtained using the highest (**a**) and lowest (**b**) sets of initial delays over the temperature range of 84–750 K.

Figure 22 shows DLTS spectra obtained from a 20 µm Ni/4H-SiC epitaxial (n-S20) SBD using a −2 to 0 V pulse with a pulse width of 1 ms on the temperature range 84 to 750 K. Figure 22a was recorded using correlator delays of 100, 50, 20, and 10 ms and Figure 22b was recorded using 0.2, 0.1, 0.05, and 0.02 ms for a shorter temperature range. The initial spectra showed four peaks; however, after gaussian deconvolution a fifth peak labeled peak #4 was observed between peaks #3 and #5. The most dominant peak is clearly peak #2.

From the Arrhenius plot (not shown), the activation energy and capture cross-section of each peak were obtained. The associated defect parameters are tabulated in Table 4. 

Peak #1 is well established to correlate with the transition of substitutional titanium (Ti) in the cubic lattice site, Ti(c), from the +3 charge state to +2 [64] and is well known to appear in SiC as a side effect of the growth process [61,64,66]. The upward bending of the left side tail of the peak for the lowest two correlators suggest the presence of the Ti(h) defect at approximately E_c_ – 0.12 eV; however, its peak was not observed in the temperature range used. Peak #2 is the Z_1/2_ center which appears in all 4H-SiC samples and has been strongly correlated to carbon vacancies as established by several annealing studies using DLTS and EPR [74,75,76]. Theoretical calculations and EPR measurements suggest that the identity of the three levels could be the (−2/0) transition of the cubic and (−1/0) transition of both the cubic and hexagonal site carbon vacancies [72,73,76,77]. Peak #3 is identified as Ci1 which is suspected to originate from chlorine impurities introduced during the growth process to compensate for silicon droplet formation [61,71]. Peaks #4 and #5 were labeled as EH_6_ and EH_7_ and are commonly grouped together as the single peak EH_6/7_ due to their close proximity [14,51,52,56,66,75,78,79,80,81]. Its concentration has a one to one correlation with Z_1/2_ suggesting its relation to the carbon vacancy. Further EPR measurements suggest that EH_6_ is the +1 donor state and EH_7_ is the +2 state [77] and this is supported by theoretical calculations as well [72,73,79]. In contrast to the n-S50 samples, the n-S20 sample did not show the presence of Ti(h), EH_5_, and the unidentified defect situated at 2.4 eV below the conduction band edge. However, n-S20 samples did show the presence of EH_6_ and EH_7_ defect levels which were not encountered in the n-S50 samples. 

#### 3.5.4. Isochronal Annealing Studies

**Figure 23:**
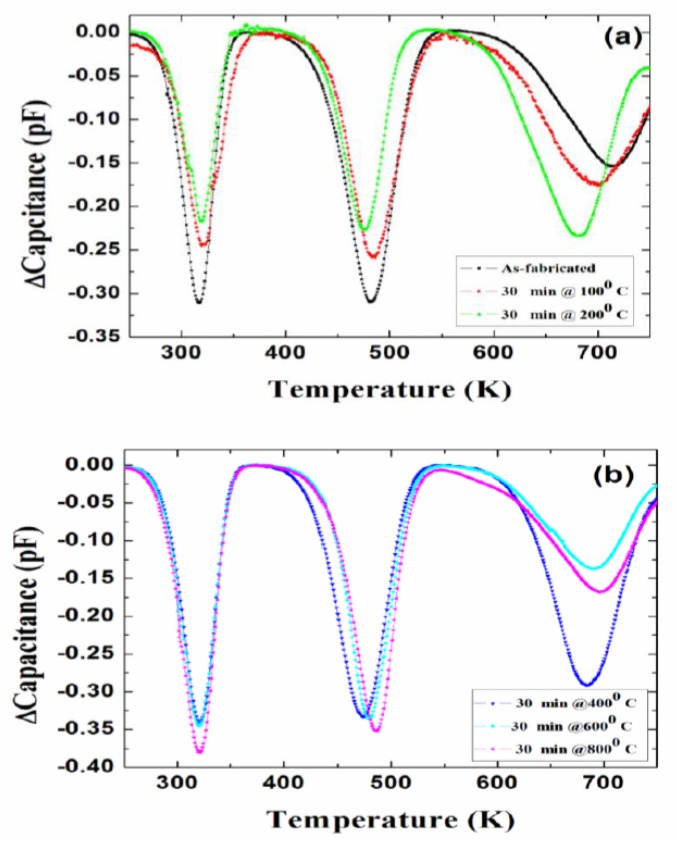
DLTS spectra obtained in a temperature range 250–750 K: (**a**) as-fabricated and annealed at 100 and 200 °C; and (**b**) annealed at 400, 600, and 800 °C.

To study the defect dynamics, i.e., transformation or disappearance of defects because of atomic motion under the influence of temperature, isochronal annealing experiments were carried out on 50 µm n-type Ni/4H-SiC (n-S50) SBDs [82]. As mentioned earlier in Section 2, the samples were annealed at a particular temperature for 30 min followed by DLTS measurements. The annealing treatments were carried out in the temperature range 100–800 °C. Figure 23 summarizes the DLTS results obtained after each annealing stage. For the sake of simplicity, the defect levels with activation energy above room temperature are shown in the figure. As is evident from the DLTS spectra, all the deep level defects were very much stable up to an annealing temperature of 800 °C. The lesser apparent defects as have been observed previously [82] are also summarized as follows. The capture cross-sections of the trap centers Ti(c), Z_1/2_, and EH_5_ were reduced by an order of magnitude when the samples were annealed at a temperature of 400 °C. The respective defect densities were observed to follow a similar trend throughout the isochronal annealing studies.

#### 3.5.5. Correlation of Detector Performance with Deep Level Defects

**Figure 24:**
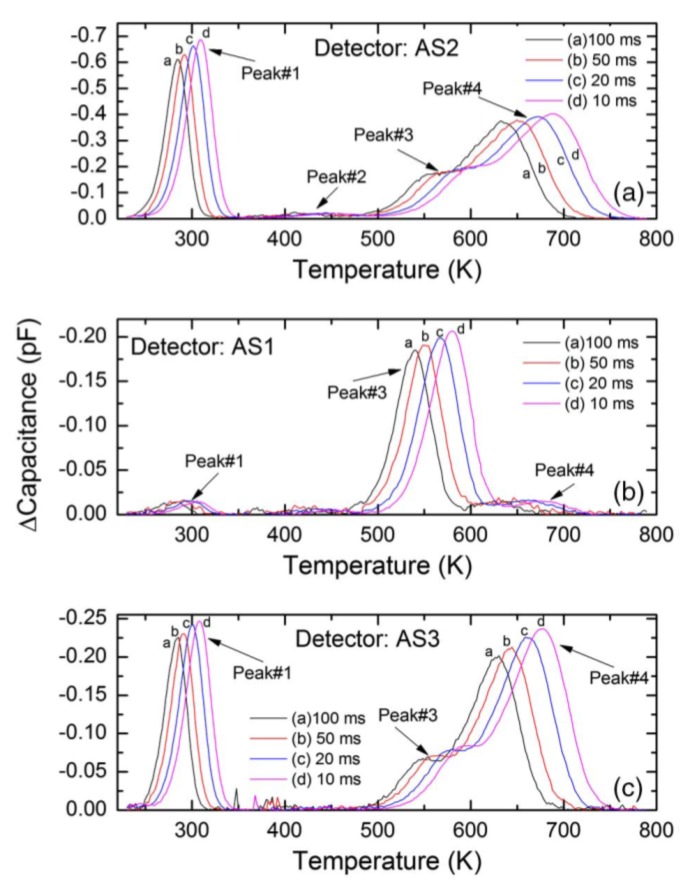
Capacitance-DLTS spectra obtained in the temperature range 250–750 K for (**a**) AS2, (**b**) AS1, and (**c**) AS3. The detectors were fabricated on n-S20 type epitaxial layers.

**Figure 25:**
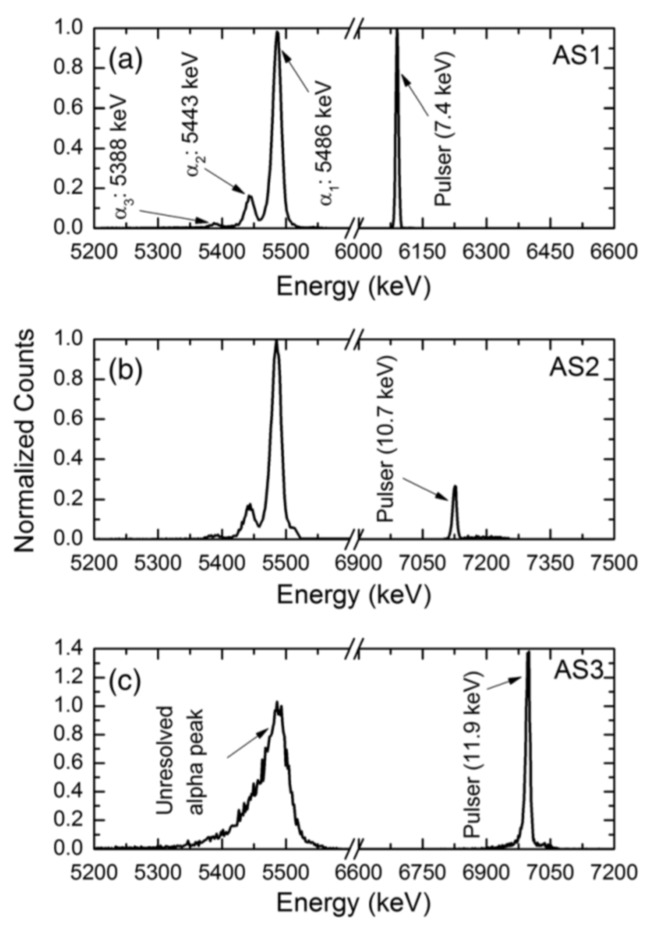
Pulse height spectra obtained using a ^241^Am point alpha source for detectors (**a**) AS1, (**b**) AS2, and (**c**) AS3.

Deep level capacitance transient spectroscopy was carried out in three n-S20 type samples. The detector performance was also studied for alpha radiation. Figure 24 shows DLTS spectra in the capacitance mode for these three samples named as AS1, AS2, and AS3. Figure 25 shows the alpha pulse-height spectra for the same detectors. The energy resolution of the detectors could be seen to vary although they were fabricated in one batch under a similar condition. The detector AS1 showed the best resolution (0.29%), followed by AS2 (0.38%) and AS3 (0.96%) for 5486 keV alpha particles. From the DLTS spectra it is evident that the best detector AS1 almost did not show Peak#1 which is associated with the Z_1/2_ defect. The trap concentration related to the other peaks were also orders of magnitude lower (except for Peak#3 which could not be properly identified) when compared to that in AS2 and AS3 [40]. Between AS2 and AS3, AS2 exhibited better detector performance which is also corroborated by the fact that the capture cross-sections of the Z_1/2_, Ci1(Peak#3), and EH_6/7_ (Peak#4) defects were at least one order of magnitude lower compared to the detector AS3. The presence of the Ci1 defect did not seem to interfere with the detector performance as the detector AS2 has much larger concentration and cross-section of the Ci1 defect compared to the other two samples. On the other hand, Z_1/2_, which was identified as carbon vacancies and situated at 0.6 eV below the conduction band edge, evidently deteriorates the performance of detectors.

Hence, although the detectors were fabricated from superior quality wafers taken from the same parent wafer and batch processed under exactly similar conditions, it can be seen that their performance as radiation detectors varied significantly. The relative difference in performance was seen to be related with the presence of defects suggesting inhomogeneous distribution of such defects in the parent wafer.

## 4. Discussion

I-V measurements on the SI epitaxial layers showed the formation of contacts with asymmetric behavior with respect to the polarity of the applied bias. These samples also showed the influence of trapping centers on the I-V patterns in the forms of steps. The n-type samples on the other hand formed very effective Schottky contacts and did not show any influence of trapping centers on the I-V curves obtained at room temperature. The typical barrier height of such Schottky contacts were found to be of the order of 1.3 eV with diode ideality factors mostly greater than 1 indicating spatial non-uniformity of barrier height. Temperature dependent I-V characteristic measurements also revealed that the effective area through which actual current flow takes place on the Ni/4H-SiC interface is at least an order of magnitude less than the actual geometric contact area.

The semi-insulating samples showed a very little capacitance (≤2 pF) and hardly showed any variation with bias voltages. The n-type epitaxial layer samples as a Schottky diode, showed a comparatively high capacitance value of typically 800 pF at 0 V reverse bias. The effective doping concentration was calculated to be of the order of 1 × 10^15^ cm^−3^, from the C-V measurements. The typical built-in potential was calculated to be 1.4 V. Surface barrier heights were also calculated from the C-V measurements and were found to be in the order of 1.47 eV which is slightly higher compared to that obtained from the I-V measurements. The reason behind this again is related to the spatial variation of barrier height which influences the I-V measurements. C-V measurements on the other hand give the average value of barrier height calculated from the capacitance involving the entire contact area. 

The quality of the SI epitaxial layers was evaluated using preferential etching and XRD rocking curve measurement techniques. The studies revealed the high quality of the epitaxial layers which showed the width of the rocking curve peak to be as low as 3.6 arc sec corresponding to the (0008) plane reflection. Theoretical calculations predicted the width of the rocking curve peak for a similar plane to be 2.7 arc sec. However, the presence of structural defects like threading screw dislocations (TSDs), threading edge dislocations (TEDs), and basal plane dislocations (BPDs) were confirmed using KOH etching and optical spectroscopy.

The electrical measurements revealed that the n-type epitaxial layers, owing to their capability of formation of Schottky diodes and less defect interference, are more suitable for detector fabrication. Hence, detectors were fabricated on n-type epitaxial layers and tested for their performance. For the radiation detection measurements mostly 20 and 50 µm superior quality n-type Ni/4H-SiC epitaxial layer SBD were used. For the calibration of our nuclear spectrometer an estimation of the electron hole-pair creation energy (EHP) was done using a method of absolute calibration. A value of ≈7.3 eV electron hole-pair creation energy was calculated and used for the subsequent measurements. Using alpha particle spectrometry, the charge collection efficiency was determined for these detectors for different applied bias voltages using which the minority (hole) carrier diffusion length in these epitaxial layers was found to be ≈18.6 µm. 

These detectors readily detected alpha particles with high efficiency even without any applied bias. Under optimized biasing and shaping conditions, an extremely high energy-resolution of ≈0.29% was achieved for 5486 keV alpha particles, without using a collimated source. Careful electronic noise analysis showed that the intrinsic detector resolution to be 10.5 keV for 5486 keV alpha particles. It was also revealed from the noise analysis that the white series noise of the spectrometer increased when the detector was plugged in and the optimized shaping time was found to shift towards 6 µs compared to 2 µs when the detector was not plugged in. These detectors also showed a very high sensitivity towards X-rays and low energy gamma rays. They also exhibited high spatial uniformity of X-ray responsivity. Pulse height spectrum revealed an energy resolution of 2.1% for 59.5 keV peak from a ^241^Am source which is comparable to that normally obtained from CdZnTe (CZT) detectors.

The subsequent studies followed on investigating the defects that control the ultimate performance of these devices. The 4H-SiC semi-insulating and good quality n-type epitaxial layers were studied using KOH etching and optical microscopy. The n-type samples showed features like comet tails, pits, hillocks, triangular defects, and step bunching, and the SI epitaxial layers showed the presence of carrot defects only. Correlation with EBIC studies on the n-type samples showed the influence of comet tail morphological defects on the current flow through the epilayers. The superior quality n-type epitaxial layers did not show the presence of etch-pits. Other sensitive techniques like TSC and DLTS measurements verified the presence of defects like Ti(h), Ti(c), Z_1/2_, EH_5_, Ci1, IL1, EH_6_, and EH_7_, with a few unidentified ones. Of all the defects, Z_1/2_ defects, which are identified as carbon vacancies and located at 0.67 eV below the conduction band edge, were seen to directly affect the detector properties most. The best detector that was tested was found to have a nominal concentration of Z_1/2_ defects compared to the rest of the detectors. Isochronal annealing studies showed that all the visible defect levels remained quite stable up to an annealing temperature of 800 °C and duration of 30 min.

## 5. Conclusions

The advent of 4H-SiC epitaxial layers and SI 4H-SiC single crystals as radiation detector materials were thoroughly studied and results were presented in terms of investigations done in our laboratories at UofSC. Our studies revealed the essential factors which regulate the performance of 4H-SiC epitaxial layer based Schottky barrier detectors. We found that the key to the performance of these devices are thin nickel contacts deposited on RCA cleaned surface which resulted in high surface barrier heights. We also observed that different sister samples obtained from the same parent wafer behave differently when compared in terms of barrier heights, ideality factors, and alpha detection resolution. It was also established that the ultimate performance of these detectors was primarily controlled by the type, concentration, and capture cross-section of the intrinsic point defects. Presence of one particular defect related to carbon vacancies, the Z_1/2_ center, was identified as the primary factor behind poor resolution of 4H-SiC epitaxial layer based SBDs.

## 6. Patents

Schottky barrier detection devices having a 4H-SiC n-type epitaxial layer, US 9,515,211 B2 (2016).

## Figures and Tables

**Table 1:** Categorization of 4H-SiC wafers used in this study.

Conductivity	Thickness (µm)	Quality	Designation
Semi-insulating	50	Good	SI-M50
n-typen	50	Good	n-M50
n-typen	50	Superior	n-S50
n-typen	20	Superior	n-S20

**Table 2:** Defect parameters acquired from TSC spectroscopy for a SI Ni/4H-SiC sample.

Peak #	T_m_ (K)	Activation Energy (eV)	Capture Cross-Section (cm^2^)	Possible Trap Identity
A	105–112	0.19	≈10^−18^	Al, B, L-center [59,61]
B	128–137	≈0.2	-	
C	236–247	0.57	≈2 × 10^−16^	Z_1/2_ [59,61,62]
D	280–290	0.82–0.87	≈10^−13^	IL_1_ [58]
E	≈525	-	-	Intrinsic defects

**Table 3:** Defect parameters acquired from DLTS spectroscopy for a 50 µm n-type Ni/4H-SiC (n-S50) epitaxial Schottky barrier detector.

Peak #	Capture Cross-Section (cm^2^)	Activation Energy (eV)	Trap Concentration (cm^−3^)	Possible Trap Identity
1	4.13 × 10^−15^	E_c_ – 0.13	1.3 × 10^13^	Ti(h) [62,64,65,66,67]
2	2.50 × 10^−15^	E_c_ – 0.17	3.6 × 10^13^	Ti(c) [62,64,65,66,67]
3	3.36 × 10^−15^	E_c_ – 0.67	1.7 × 10^13^	Z_1/2_ [66,67,68,69]
4	3.73 × 10^−15^	E_c_ – 1.04	2.1 × 10^13^	EH_5_ [70,71,72,73]
5	3.22 × 10^−17^	E_c_ – 1.30	7.9 × 10^12^	Ci1 [71]
6	1.53 × 10^−11^	E_c_ – 2.40	5.6 × 10^12^	Unidentified

**Table 4:** Defect parameters acquired from the DLTS spectra for a 20 µm n-type Ni/4H-SiC epitaxial (n-S20) Schottky barrier detector.

Peak #	Capture Cross-Section (cm^2^)	Activation Energy (eV)	Trap Concentration (cm^−3^)	Possible Trap Identity
Peak #1	1.742 × 10^−14^	E_c_ − 0.170	2.05 × 10^12^	Ti(c)
Peak #2	3.970 × 10^−15^	E_c_ − 0.647	2.67 × 10^12^	Z_1/2_
Peak #3	1.721 × 10^−14^	E_c_ − 1.385	4.89 × 10^11^	Ci1
Peak #4	1.055 × 10^−15^	E_c_ − 1.316	6.39 × 10^11^	EH_6_
Peak #5	2.672 × 10^−15^	E_c_ − 1.537	1.21 × 10^12^	EH_7_
